# Supplementary material for: Long non-coding RNAs direct the SWI/SNF complex to cell type-specific enhancers
Source: Nat Commun. 2025 Jan 2;16:131. doi: 10.1038/s41467-024-55539-6 (PMC11695977; doi:10.1038/s41467-024-55539-6)
Supplement: Supplementary file 1 — Supplementary Information [file 41467_2024_55539_MOESM1_ESM.pdf]

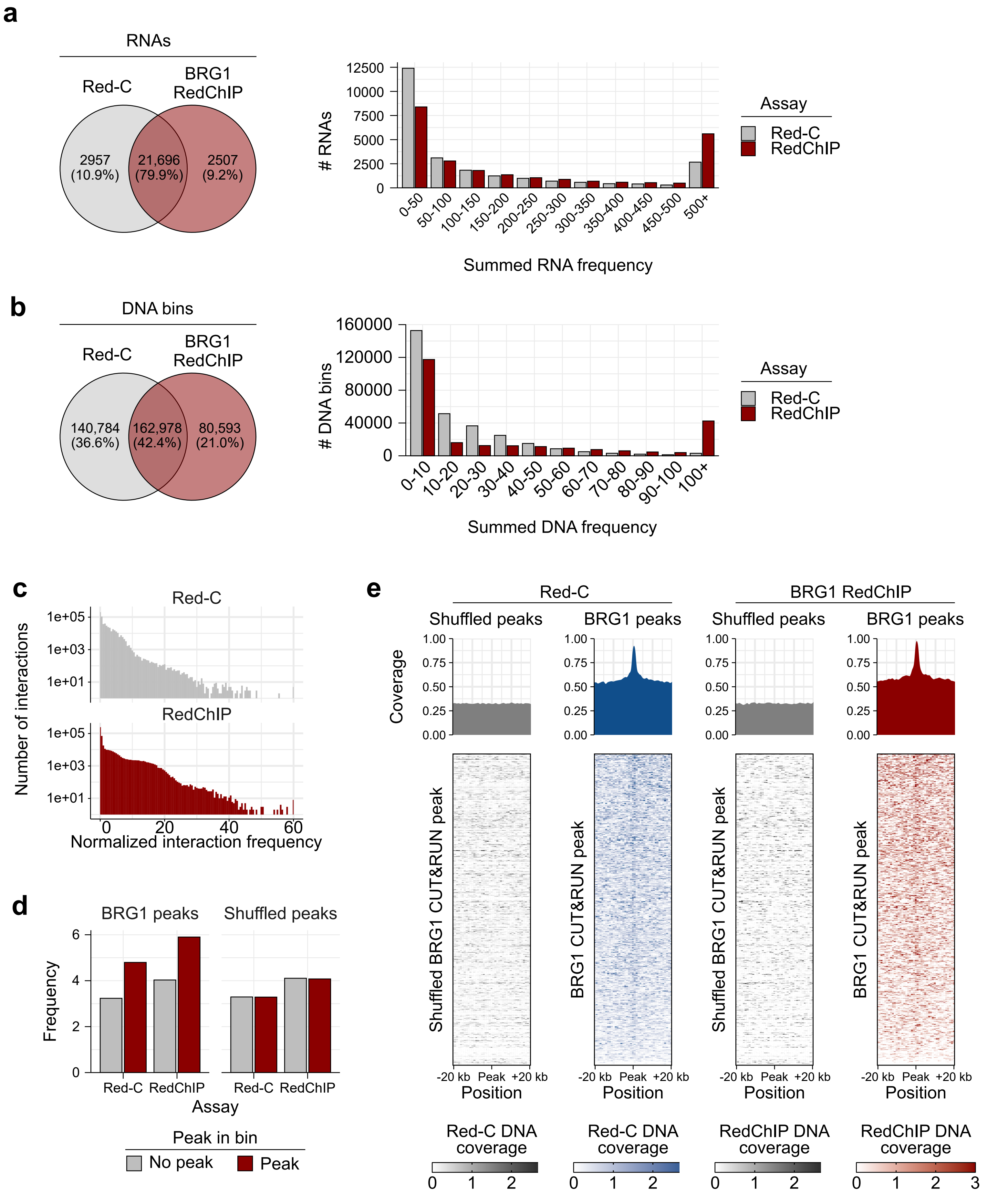

Supplementary Fig. 1 | BRG1 RedChIP and CUT&RUN

(a) Overlap of RNAs between Red-C and BRG1 RedChIP and plotted individually across summed RNA frequencies.
(b) Overlap of DNA bins between Red-C and BRG1 RedChIP and plotted individually across ranges of summed DNA frequencies.
(c) Red-C and BRG1 RedChIP coverage. Number of RNA-DNA interactions across the normalized interaction frequencies.
(d) Mean DNA bin interaction frequency in Red-C and BRG1 RedChIP data at BRG1 CUT&RUN peaks versus a size-matched set of shuffled peaks.
(e) Genome-wide density plots for RNA-DNA interactions for both Red-C and RedChIP DNA coverage at BRG1 CUT&RUN peaks versus a size-matched set of shuffled peaks.

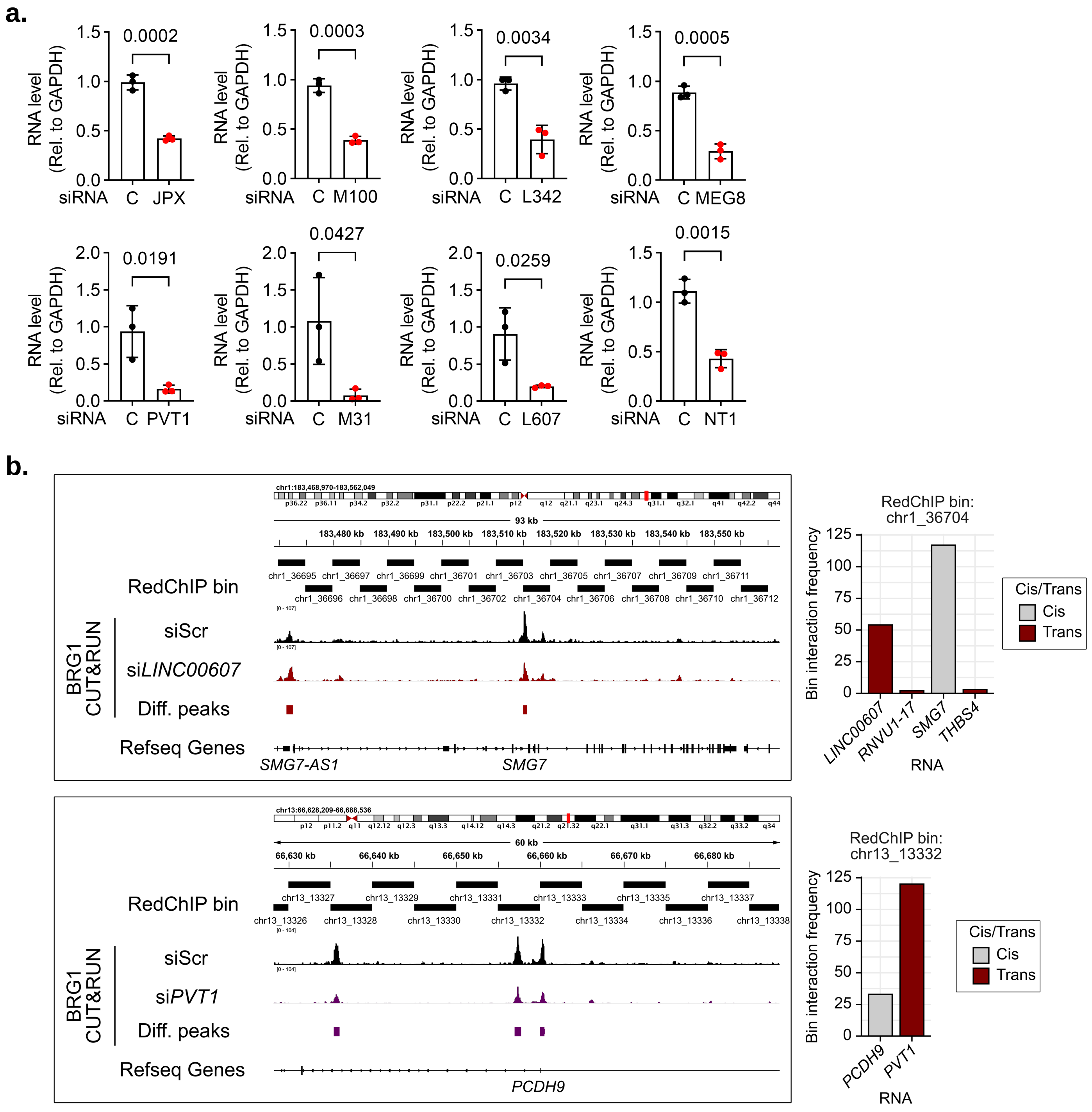

Supplementary Fig. 2 | RT-qPCR validation of knockdowns and knockdowns on *cis/trans* RNA binding sites

(a) siRNA against *JPX*, *MIR100HG* (M100), *LINC00342* (L342), *MEG8*, *PVT1*, *MIR31HG* (M31), *LINC00607* (L607) and *NEAT1* (NT1) followed by RT-qPCR. RNA level relative to GAPDH RNA displayed as mean +/- SD, n=3, unpaired t-test. *P*-values are indicated above each bar plot.

(b) Browser traces demonstrating BRG1 CUT&RUN peaks at mixed cis/trans RNA binding sites. Depletion of the trans RNA e.g. LINC00607 (top panel) and PVT1 (bottom panel) reduces BRG1 binding at the genomic locus of the cis RNA (SMG7 and PCDH9 respectively). Graphs on the right demonstrate the degree of binding for each RNA at the respective mixed cis/trans binding site.

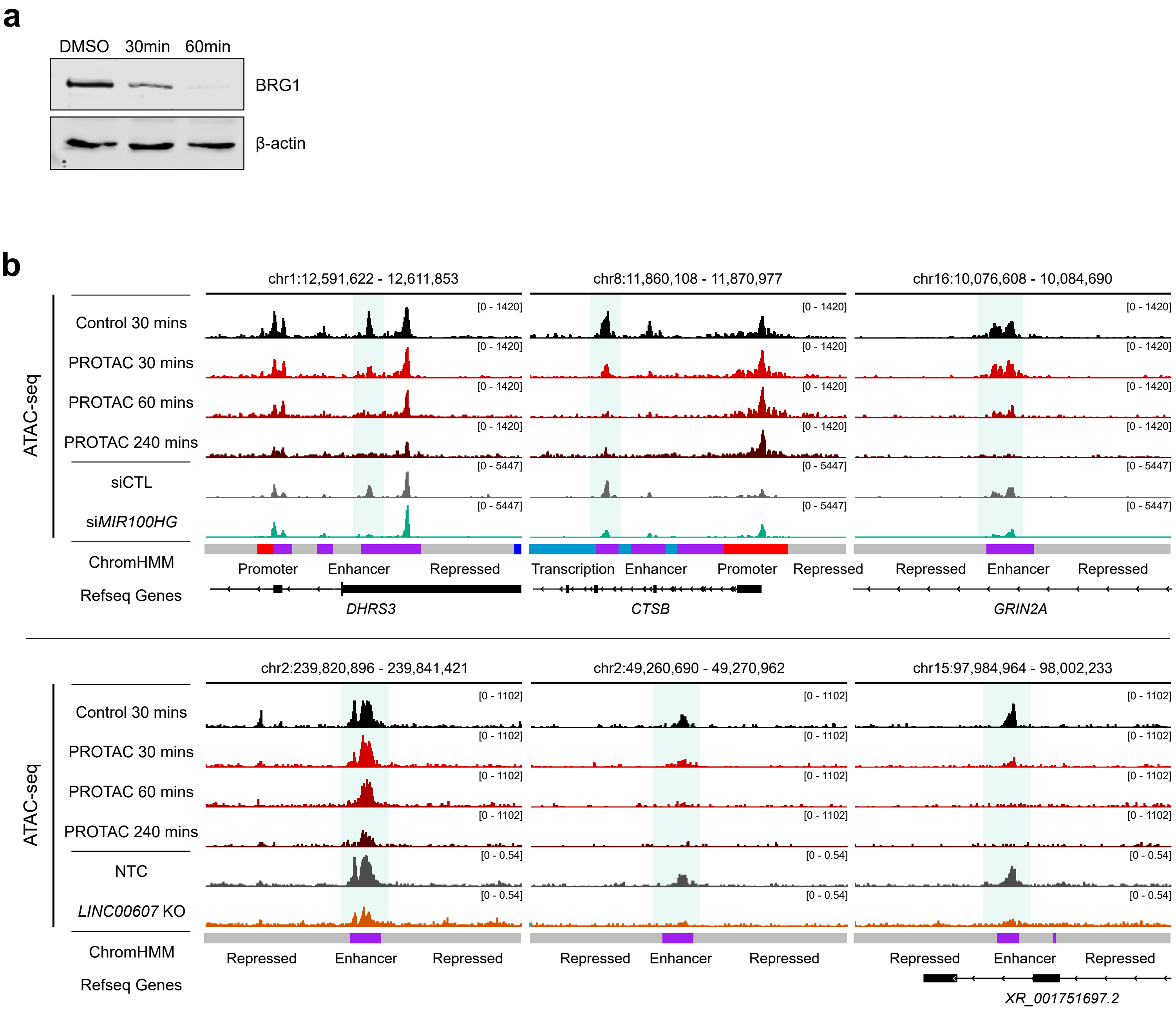

Supplementary Fig. 3 | PROTAC AU-15330 on BRG1 levels and ATAC-seq

(a) Western blot of HUVEC treated with 1 µM AU-15330 PROTAC (30 and 60 min) or DMSO (60 min). Antibodies against BRG1 and β-actin. Blot representative of 3 biological replicates with each giving similar results.
(b) ATAC-seq following treatment of HUVEC with 1 µM AU-15330 PROTAC (30, 60 or 240 min) or DMSO (30 min), with control or *MIR100HG* siRNAs (top), and non-targeting control (NTC) or *LINC00607* CRISPR-Cas9 (bottom).

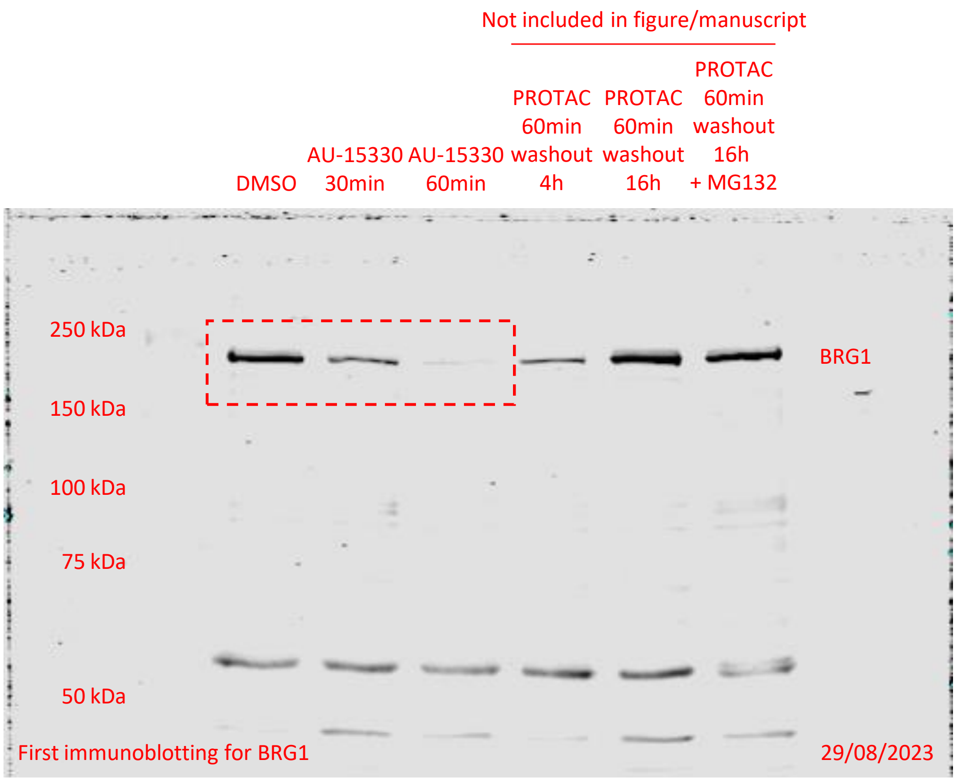

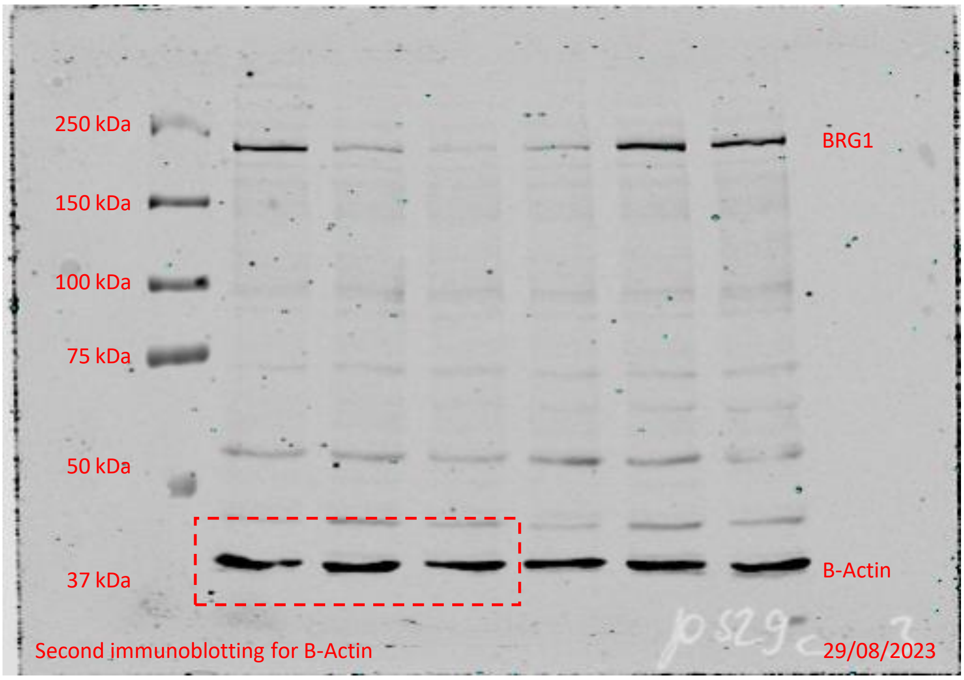

Uncropped blots for Supplementary Fig. 3a.
