# Supplementary material for: Long non-coding RNAs direct the SWI/SNF complex to cell type-specific enhancers
Source: Nat Commun. 2025 Jan 2;16:131. doi: 10.1038/s41467-024-55539-6 (PMC11695977; doi:10.1038/s41467-024-55539-6)
Supplement: Supplementary file 3 — Reporting Summary [file 41467_2024_55539_MOESM3_ESM.pdf]

nature portfolio

Corresponding author(s): Matthias S. Leisegang

Last updated by author(s): Oct 21, 2024

Reporting Summary

Nature Portfolio wishes to improve the reproducibility of the work that we publish. This form provides structure for consistency and transparency in reporting. For further information on Nature Portfolio policies, see our Editorial Policies and the Editorial Policy Checklist.

Statistics

For all statistical analyses, confirm that the following items are present in the figure legend, table legend, main text, or Methods section.

n/a	Confirmed	
☐	☒	The exact sample size (*n*) for each experimental group/condition, given as a discrete number and unit of measurement
☐	☒	A statement on whether measurements were taken from distinct samples or whether the same sample was measured repeatedly
☐	☒	The statistical test(s) used AND whether they are one- or two-sided<br>*Only common tests should be described solely by name; describe more complex techniques in the Methods section.*
☐	☒	A description of all covariates tested
☐	☒	A description of any assumptions or corrections, such as tests of normality and adjustment for multiple comparisons
☐	☒	A full description of the statistical parameters including central tendency (e.g. means) or other basic estimates (e.g. regression coefficient) AND variation (e.g. standard deviation) or associated estimates of uncertainty (e.g. confidence intervals)
☐	☒	For null hypothesis testing, the test statistic (e.g. *F*, *t*, *r*) with confidence intervals, effect sizes, degrees of freedom and *P* value noted<br>*Give P values as exact values whenever suitable.*
☒	☐	For Bayesian analysis, information on the choice of priors and Markov chain Monte Carlo settings
☐	☒	For hierarchical and complex designs, identification of the appropriate level for tests and full reporting of outcomes
☒	☐	Estimates of effect sizes (e.g. Cohen's *d*, Pearson's *r*), indicating how they were calculated

Our web collection on statistics for biologists contains articles on many of the points above.

Software and code

Policy information about availability of computer code

Data collection	No software was used.
Data analysis	AriaMX qPCR software (Agilent Aria v1.7), Licor Image Studio (v5.2), Integrative Genomics Viewer (IGV) (v2.16.2, 14/07/2023), Bowtie2 (v2.4.5), FastQC (v0.11.8), FASTX-Toolkit (v0.0.14), seqtk (version 1.3), Flexbar (version 3.4.0), Samtools (v1.9 for iCLIP, v1.10 for RedChIP and ATAC-seq), bedtools (v2.27.1), STAR (v2.7.3a for iCLIP and RNA-Seq, v2.7.10 for RedChIP), PureCLIP (v1.3.1), BindingSiteFinder (v1.0.0), FastUniq (v1.1), Trimmomatic (v0.39), SeqKit (v2.7.0), cutadapt (v2.8), Homer (v4.1.1), DESeq2 (v1.26.0), bamCoverage (v3.5.3), MACS3 (v3.0.0), featureCounts v1.6.5, DiffBind (v3.10.1), ChromHMM (v1.24), STARE (v1.0), GraphPad Prism 10.1.2, Gencode (v43), Ensembl human genome version hg38 (release 99)

For manuscripts utilizing custom algorithms or software that are central to the research but not yet described in published literature, software must be made available to editors and reviewers. We strongly encourage code deposition in a community repository (e.g. GitHub). See the Nature Portfolio guidelines for submitting code & software for further information.

Data

Policy information about availability of data

All manuscripts must include a data availability statement. This statement should provide the following information, where applicable:

- Accession codes, unique identifiers, or web links for publicly available datasets
- A description of any restrictions on data availability
- For clinical datasets or third party data, please ensure that the statement adheres to our policy

All NGS datasets have been deposited and are available at NCBI GEO with the accession number GSE262070 at the following URL: https://www.ncbi.nlm.nih.gov/geo/query/acc.cgi?acc=GSE262070
ATAC-seq of LINC00607 KO and NTC is publicly available at NCBI GEO with the accession number GSE199878.

Research involving human participants, their data, or biological material

Policy information about studies with human participants or human data. See also policy information about sex, gender (identity/presentation), and sexual orientation and race, ethnicity and racism.

Reporting on sex and gender	Human biological material used in this study were only commmercially available eukaryotic cells. These were pooled human umbilical vein endothelial cells (HUVEC), purchased from PromoCell (C-12203, Lot No. 405Z013, 408Z014, 416Z042, Heidelberg, Germany). The HUVEC batches originate from umbilical cord/ umbilical vein of caucasians (405Z013: 2 males, 1 female; 408Z014: 2 males, 1 female; 416Z042: 2 males, 2 females).
Reporting on race, ethnicity, or other socially relevant groupings	Not used in this study.
Population characteristics	Not used in this study.
Recruitment	Not used in this study.
Ethics oversight	Not used in this study.

Note that full information on the approval of the study protocol must also be provided in the manuscript.

Field-specific reporting

Please select the one below that is the best fit for your research. If you are not sure, read the appropriate sections before making your selection.

[x] Life sciences [ ] Behavioural & social sciences [ ] Ecological, evolutionary & environmental sciences

For a reference copy of the document with all sections, see nature.com/documents/nr-reporting-summary-flat.pdf

Life sciences study design

All studies must disclose on these points even when the disclosure is negative.

Sample size	No formal statistical sample size calculation was performed. Sample sizes were selected based on previously published studies in the field that demonstrated significant findings with similar group sizes. PMID: 34969862, RedChIP (Gavrilov et al. 2022); PMID: 21559008, iCLIP (Konig et al. 2011); PMID: 28079019, CUT&RUN (Skene and Henikoff 2017); PMID: 25559105, ATAC-seq (Buenrostro et al. 2015)).<br>For in vitro knockdown experiments, at least three independent biological replicates were conducted to ensure reproducibility and to identify significant differences between conditions. Red-type experiments were performed with one biological replicate, with Red-C serving as the background control and subsequent overlay with respective CUT&RUN experiments performed in triplicate. Red-type experiments involve extensive technical and quality control steps and are expensive to sequence. CLIP experiments were performed with 2 technical replicates and compared with an additional 2 technical replicates with higher UVC exposure. Taken together, this allowed for the identification of high confidence binding sites.
Data exclusions	No data was excluded.
Replication	All results were reproduced in at least three independent replicates including all successful attempts; sequencing data experiments were performed as indicated.
Randomization	Not used.
Blinding	All sequencing and analysis of NGS experiments was performed blinded. Blinding was not possible for some wet lab experiments.

Reporting for specific materials, systems and methods

We require information from authors about some types of materials, experimental systems and methods used in many studies. Here, indicate whether each material, system or method listed is relevant to your study. If you are not sure if a list item applies to your research, read the appropriate section before selecting a response.

Materials & experimental systems

n/a	Involved in the study
[ ]	[x] Antibodies
[ ]	[x] Eukaryotic cell lines
[x]	[ ] Palaeontology and archaeology
[x]	[ ] Animals and other organisms
[x]	[ ] Clinical data
[x]	[ ] Dual use research of concern
[x]	[ ] Plants

Methods

n/a	Involved in the study
[x]	[ ] ChIP-seq
[x]	[ ] Flow cytometry
[x]	[ ] MRI-based neuroimaging

Antibodies

Antibodies used	Anti-BRG1 (A303-877A , Bethyl), anti-BRG1 [EPNCIR111A] (ab110641, Abcam), anti-β-actin (A1978, Sigma Aldrich).<br>Anti-BRG1 (ThermoFisher, A303-877A) and Anti-β-Actin (Sigma Aldrich, A1978) were used for Western blotting at dilutions of 1:1000 and 1:2000 respectively. For CUT&RUN experiments, 1 µL of anti-BRG1 (Abcam, ab110641) was used for each sample. 4 µL was used for RedChIP. 6 µg were used for CLIP experiments.
Validation	Prior to use, the BRG1 antibodies were validated in BRG1 CRISPR/Cas9 KO cells. All antibodies were purchased from commercial vendors, who provide validation information on their website.<br>Anti-BRG1 (A303-877A , Bethyl): https://www.thermofisher.com/antibody/product/BRG1-SMARCA4-Antibody-Polyclonal/A303-877A<br>anti-BRG1 [EPNCIR111A] (ab110641, Abcam): https://www.abcam.com/en-de/products/primary-antibodies/brg1-antibody-epncir111a-ab110641#<br>anti-β-Actin: (A1978, Sigma Aldrich): https://www.sigmaaldrich.com/DE/en/product/sigma/a1978

Eukaryotic cell lines

Policy information about [cell lines and Sex and Gender in Research]

Cell line source(s)	Pooled human umbilical vein endothelial cells (HUVEC) were purchased from PromoCell (C-12203, Lot No. 405Z013, 408Z014, 416Z042, Heidelberg, Germany). The HUVEC batches originate from umbilical cord/ umbilical vein of caucasians (405Z013: 2 males, 1 female; 408Z014: 2 males, 1 female; 416Z042: 2 males, 2 females).
Authentication	Prior to use, HUVECs were tested for endothelial cell marker expression, their angiogenic potential, their response to VEGF-A, laminar shear stress and inflammatory treatments. Transcriptomic analysis (RNA-seq) and chromatin accessibility assays (ATAC-seq) of the cells were also performed and correlated to other published HUVEC data.
Mycoplasma contamination	All cells used in this study tested negative for Mycoplasma contamination.
Commonly misidentified lines (See ICLAC register)	No commonly misidentified lines were used.

Plants

Seed stocks	No plants were used in this study.
Novel plant genotypes	No plants were used in this study.
Authentication	No plants were used in this study.
